# Clinical Failures of Linezolid and Implications for the Clinical Microbiology Laboratory

**DOI:** 10.3201/eid0812.020139

**Published:** 2002-12

**Authors:** Brian A. Potoski, Julie E. Mangino, Debra A. Goff

**Affiliations:** * Ohio State University Medical Center, Columbus, Ohio, USA

Linezolid is the first in a new class of antimicrobials, the oxazolidinones. This antimicrobial is approved to treat gram-positive infections caused by vancomycin-resistant enterococci (VRE) and methicillin-resistant *Staphylococcus aureus* (MRSA) (1). Newer antibiotics have provided clinicians greater treatment options; however, ongoing experience shows limitations in their use. We report two patients with infections caused by linezolid-resistant and possible linezolid-nonsusceptible bacteria and provide commentary on the increasing bacterial resistance to linezolid and quinupristin/dalfopristin, as well as the appropriate use of these and future antibiotics.

## Patient 1

A 47-year-old man’s history was notable only for right ankle and subtalar arthritis. He underwent right ankle fusion with a nail placement in October 2000. In April 2001, he went to the emergency department for ankle pain secondary to a newly displaced fracture. The fusion nail was removed in the operating room; placement of a new longer nail was uncomplicated. One month later, he developed an ankle hematoma; it was drained, and he was given cephalexin for 7 days. Over the next several weeks, the area became erythematous and swollen; it spontaneously drained purulent material. The patient underwent irrigation and debridement on May 31, 2001. Gross pus was obtained and sent for Gram stain and culture. The patient was discharged with a prescription for amoxicillin/clavulanate tablets.

Culture grew *S. aureus* susceptible to vancomycin, trimethoprim/sulfamethoxazole (TMP-SMX), and gentamicin and resistant to all beta-lactams and clindamycin. Beta-lactams tested included penicillin, nafcillin, amoxicillin/clavulanic acid, cefazolin, and imipenem. Susceptibility testing of this MRSA isolate to linezolid was not performed. All testing was conducted by using a MicroScan (Dade Behring, Inc., West Sacramento, CA) automated system (unless otherwise noted) in accordance with National Committee on for Clinical Laboratory Standards (NCCLS) testing and quality-control recommendations (2).

Amoxicillin/clavulanic acid was discontinued, and oral linezolid, 600 mg twice a day, was begun. The patient did well, progressing to full weight-bearing capacity, and finished a 7-week course of linezolid. Two days after completing the course, nausea, fever, and chills developed, and he resumed linezolid. He went to the emergency room on July 30 with a temperature of 103°F; his ankle was warm and tender to palpation. Linezolid was stopped, and intravenous vancomycin, 1 g every 12 hrs, was started. The nail was surgically removed, Gram stain and cultures were obtained, and a peripherally inserted central catheter line was placed intraoperatively. He was continued on vancomycin. Cultures grew MRSA with the same sensitivities as the previous MRSA isolate. Additional susceptibility testing of this isolate to linezolid and TMP-SMX by E-test was performed; both were susceptible (MIC=4 μg/mL and 0.047 μg/mL, respectively). After 4 weeks, vancomycin was discontinued, and oral TMP-SMX , two double-strength tablets twice daily, was initiated for an additional 8 weeks of therapy. At followup, the patient’s laboratory results showed a decreased leukocyte count, erythrocyte sedimentation rate, and C-reactive protein. He subsequently finished a total of 12 weeks of therapy and remained asymptomatic 6 months after completion of treatment.

## Patient 2

A 41-year-old woman with refractory acute lymphocytic leukemia was admitted in May 2001 for an allogeneic bone marrow transplant. Her hospital course was complicated by *Klebsiella pneumoniae* sepsis, neutropenia, mental status changes, acute renal failure, and respiratory distress. She received several courses of antibiotics for extended periods including imipenem, amikacin, piperacillin/tazobactam, vancomycin, amphotericin B lipid complex, fluconazole, ciprofloxacin, and tobramycin. While she was receiving vancomycin, both peripheral and central venous catheter blood cultures grew vancomycin-resistant *Enterococcus faecium* (MIC >16 μg/mL)*.* The isolate was also resistant to other antibiotics tested (MIC of VRE was >8 μg/mL for both ampicillin and penicillin). Vancomycin was discontinued, intravenous linezolid, 600 mg every 12 hrs, was begun, and susceptibility testing to linezolid was requested. She remained on linezolid and died 3 days later. Subsequent susceptibility results revealed the isolate to be resistant to linezolid by E-test (MIC = 32 μg/mL). Confirmatory testing by broth dilution and E-test was performed at a reference lab, which verified resistance to linezolid and intermediate susceptibility to quinupristin/dalfopristin (MIC = 2 μg/mL).

Patient 1, a pharmacist, preferred the convenience of oral linezolid therapy to intravenous vancomycin. Initial susceptibility of the MRSA isolate to linezolid was not obtained; however, a previous case report described success in treating MRSA and VRE bacteremia in a patient with MRI (magnetic resonance imagery)–confirmed vertebral osteomyelitis as the primary focus for infection (3). Although the second MRSA isolate in our patient was considered susceptible to linezolid by the NCCLS-defined MIC interpretive standard (MIC ≤4 μg/mL), the patient did not respond to a 7-week course of linezolid. Of interest, the MIC of MRSA to linezolid from our patient’s isolate was 4 μg/mL, three dilutions greater than the isolate in the prior case report (MIC=0.5 μg/mL).

Patient 2’s treatment was changed from empiric vancomycin to linezolid for VRE bacteremia without linezolid being part of her initial susceptibility data. Only after the patient died did the requested sensitivities to linezolid became available. Had linezolid been part of the initial susceptibility data, other therapies most likely would have been pursued since the isolate was resistant to linezolid. Arguably, her death might have been averted.

Multidrug-resistant organisms, especially VRE, continue to contribute to illness and death (4,5). In a published case series involving linezolid for 15 patients with VRE infections, mortality was noted to be very high at long-term followup (6). Two explanations might account for this high rate. First, the patients identified were severely ill with several coexisting conditions or illnesses. Second, other options were pursued before the initiation of linezolid, which may have inadvertently contributed to increased illness and death because of the delay in giving linezolid therapy. This latter argument is supported by studies (7,8) that show a significantly greater increase in hospital deaths if patients received inappropriate antimicrobial therapy compared to those whose initial therapy was appropriate.

These data, in addition to these recent experiences, have led us to routinely conduct susceptibility testing of linezolid and quinupristin/dalfopristin to VRE obtained from any sterile site. Previously, susceptibility data of linezolid and quinupristin/dalfopristin to VRE or MRSA were not routinely conducted unless specifically requested. Once the standard susceptibility panel was reported, if additional susceptibility tests were requested, they would be conducted with the E-test (AB Biodisk North America, Inc., Piscataway, NJ). This procedure was mainly because these drugs were not yet included on microtiter plates for commonly used automated systems (e.g., MicroScan, Vitek), and clinical reports of resistance were scarce. In addition, clinicians have a rather limited window of opportunity to request susceptibility testing to these newer agents because most bacterial isolates are saved for only 5 days. Our current practice includes obtaining tests of a VRE isolate’s susceptibility to linezolid and quinupristin/dalfopristin as routine practice if the isolate is obtained from any sterile site. As experience and reports of resistance increase, this practice may guide appropriate therapy. Because of the large number of *S*. *aureus* isolates resistant to methicillin submitted to the microbiology laboratory annually, and because linezolid nonsusceptibility to clinical isolates of MRSA has only been reported once (9), we continue to conduct susceptibility testing of linezolid and quinupristin/dalfopristin to MRSA isolates only when requested.

To make the most appropriate use of these newer antimicrobial agents, data on susceptibility are needed. We recommend that tests for susceptibility to linezolid and quinupristin/dalfopristin be conducted before treatment is initiated. Although this procedure may no longer be necessary once standardized microtiter plates include these antibiotics, it is nevertheless relevant as clinical failures and reports of resistance mount. (4,9,10) Assuming antimicrobial sensitivity because an antibiotic is a recent addition to the antimicrobial armamentarium may lead to increased illness, deaths, and costs.
